# Your Relevance Feedback Is Essential: Enhancing the Learning to Rank Using the Virtual Feature Based Logistic Regression

**DOI:** 10.1371/journal.pone.0050112

**Published:** 2012-12-10

**Authors:** Fei Cai, Deke Guo, Honghui Chen, Zhen Shu

**Affiliations:** Science and Technology on Information Systems Engineering Laboratory, National University of Defense Technology, Changsha, China; University of Illinois-Chicago, United States of America

## Abstract

Information retrieval applications have to publish their output in the form of ranked lists. Such a requirement motivates researchers to develop methods that can automatically learn effective ranking models. Many existing methods usually perform analysis on multidimensional features of query-document pairs directly and don't take users' interactive feedback information into account. They thus incur the high computation overhead and low retrieval performance due to an indefinite query expression. In this paper, we propose a Virtual Feature based Logistic Regression (VFLR) ranking method that conducts the logistic regression on a set of essential but independent variables, called virtual features (VF). They are extracted via the principal component analysis (PCA) method with the user's relevance feedback. We then predict the ranking score of each queried document to produce a ranked list. We systematically evaluate our method using the LETOR 4.0 benchmark datasets. The experimental results demonstrate that the proposal outperforms the state-of-the-art methods in terms of the Mean Average Precision (MAP), the Precision at position k (P@k), and the Normalized Discounted Cumulative Gain at position k (NDCG@k).

## Introduction

Ranking the tremendous candidate documents in accordance with the relevance to a query is an essential problem in the field of Information Retrieval (IR). That is, given a query, all documents in a document repository are sorted according to their relevance to the query by their ranking scores. A list of top ranked documents are then responded to the user. Ideally, the highest relevant document must be on the top while the least matching document appears at the bottom. The ranking method is always an active research topic and is widely used in the recommender system, machine translation, question answering and other areas.

The key insight behind the related ranking research is to develop a ranking model or a ranking function that measures the relevance of a query and documents. Several empirical ranking models have been proposed, such as the Extended Boolean Model [1], the vector space model [2], BM25 [3] and the language model [4]. Such ranking models suffer the difficulty of empirically tuning their parameters. To address such a problem, machine learning techniques, called the learning-to-rank methods, have been proposed to construct the ranking models automatically [5]–[7]. By leveraging labeled query-document pairs with their relevance and the machine learning algorithms, these approaches are able to make the parameter tuning of ranking model be more effective. Actually, many commercial search engines have widely adopted the methods using the machine learning techniques [8].

Other efforts have been done on associating the traditional ranking models with the machine learning algorithms [9]–[12]. Many representative algorithms are proposed to minimize a loss function and maximize the accuracy of a ranking model in terms of an IR metric [13]. In this paper, we propose an alternative ranking algorithm, called Virtual Feature based Logistic Regression (VFLR), which utilizes the user's relevance feedback. Instead of optimizing a specific metric, e.g., MAP, Precision and NDCG, our VFLR method generates a regression model, which presents a set of training pairs by means of virtual features extracted by the PCA and obtains a weight coefficient set of related features by the logistic regression to gain the final relevance score. These independent features don't have any realistic meanings, but convey some hidden information of the visible ones. That's why we call them as virtual features.

In the VFLR method, we assume that the user's relevance feedback on query-document pairs is always correct. Actually, for a normal user who retrieves information from Internet, he can always determine which responded documents are relevant although he may not express his requirement as exactly as a specialist. Based on this assumption, a regression model is built by the VFLR algorithm. Then the regression coefficients of the model are directly used to estimate the relevance score of documents in the test set.

The process of achieving satisfied results may involve rounds of interactive actions due to the following reasons. It may be difficult for users to formulate a good query when their requirements are not very straightforward. Thus, it makes sense for users to engage in the iterative feedback operation for the purpose of deriving more appropriate results via the automatic query term expansion or query term weighting of retrieval systems. Besides, relevance feedback can also be effective in tracking a user's evolving requirement. Users may revise their requirements after achieving some responses. Image search [14] provides a good example of the relevance feedback, where users always fail to formulate their requirements in words, but can easily judge they are relevant or non-relevant responded images. However, in the VFLR approach, only one round is performed due to the reasons: (a) other rounds are repetitions with the same principle of the round one; (b) after the round one, the performance has been improved successfully, which can validate the correctness of our proposal. Compared to other information retrieval methods that ignore the feedback from users, the VFLR algorithm overcomes the problem that a bad initial query incurs dissatisfactory retrieval results and extensive time to review a large number of responded documents for identifying the required ones.

The major reasons why the VFLR algorithm outperforms prior methods are as follows. First, the VFLR approach is conducted by leveraging the most useful information of the multidimensional document features. Second, the regression analysis is combined with user's relevance feedback. Prior works may only concentrate on user's relevance feedback to expand the initial query, but don't incorporate it into the regression analysis. The experimental results have demonstrated the superiority of the VFLR method compared to prior baselines. In summary, we give both theoretical justification and empirical verification for the VFLR method. Specifically, we validate it on the real world datasets.

According to literature [7], the current learning-to-rank methods can be divided into three categories: (a) point-wise, (b) pair-wise and (c) list-wise approaches, whose training data are individual documents, document pairs and document lists, respectively. In the case of point-wise approaches [15], training data is composed of single document. The learning process tries to project document features into relevance estimation of query-document pairs while the individual test document is assigned with unique scores according to different learned models. The output is a list of documents in descending order of scores. Some regression models can be applied to construct point-wise ranking models. Ramesh [5] adopts the Maximum Entropy (ME) regression model to build a ranking model. A fast gradient descent algorithm is used to obtain the weights of all document features. An obvious disadvantage is that no dependence between training documents is considered. Such a drawback can be partly addressed by the pair-wise models. In the case of pair-wise approaches, document pairs and the preference relation among them constitute the learning data. The problem of learning-to-rank is thus formalized as the classification problem. Literature [16] proposes a ranking method based on SVM, called as RankSVM. RankSVM takes the difference between any document pairs into account and develops a class of linear ranking functions. Burges [6] presents the RankNet model based on Neural Nets. The training procedure is to minimize the differences between the expected rank and the realistic rank produced by models via tuning parameters. GBRank [17] is similar to RankSVM, but it uses a quadratic penalization and is combined with functional gradient boosting. Although the dependence between any document pair has been considered, the dependence in the whole rank hasn't been fully considered. For the point-wise and pair-wise approaches, the positional information is invisible to their loss functions, and they both ignore the fact that some documents (or document pairs) are associated with the same query. Comparatively speaking, the list-wise approach takes the entire set of documents associated with a query in the training data as the input and predicts their ground truth relevance labels to produce their ranked list as the output. Although there has been relatively little work on the list-wise method, it seems to be the most promising one among the three methods. Xu [18] presents the AdaRank model with the loss function based on the IR performance estimation measure. The optimum parameters are determined during the learning procedure that is much relatively complex. Qin [19] presents the RankCosine model to improve the ranking precision inspired by the RankBoost model and the Vector Space model.

Meanwhile, Geng et al [20] employ different ranking models for different queries and conduct the query-dependent ranking. They propose a K-Nearest Neighbor (KNN) method for query-dependent ranking by using the labeled neighbors of the query in the query feature space and then ranking the documents with respect to the query using the created model. Veloso et al [21] develop a novel method that exploits rules in the training phase. It associates document features with its relevance to the query, and then uses the discovered rules to estimate the relevance score for ranking documents. Bennett et al [22] present a simple framework for classification-enhanced ranking that uses clicks in combination with the classification of web pages to derive a class distribution for the query. Furthermore, it uses the new defined class features to rank.

In this paper we are also interested in the learning-to-rank method. We exploit statistical information of query-document pairs with the user's relevance feedback, and then estimate the relevance of query-document pairs at query-time. The proposed method differs significantly from existing ones that are traditionally based on the entire stable document features without considering the user's relevance feedback. The relevance prediction of query-document pair by the stable regression model is very reliable, and the final ranked list is outperforming. Our approach is relatively simple but extremely effective, as we will show in the latter experiments.

## Methods

The task of learning-to-rank in IR area is defined as follows. We use the training dataset (referred as 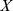) as input, which consists of a set of records by the form 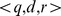, where 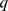 is a query (represented as a list of terms {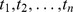}), 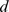 is a document (represented as a list of features 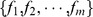), such as term frequency (TF), inverse document frequency (IDF) and document length (DL) of the whole document, and 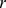 is the relevance of 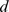 to 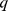. The value of 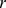 can be one of a set of levels, e.g., 0, 1, 2, 3 and 4. The training dataset is used to construct a deterministic model based on the document features and their relevance to related queries. The test set (referred as 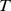) consists of records 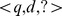, where only the query and the document features are known while the relevance of 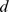 to 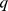 is unknown. The model learned from the training phase is utilized to estimate the unknown relevance score of a document to a query, which can be further used to generate a final ranked list.

We propose a Virtual Feature based Logistic Regression (VFLR) method for the relevance estimation of query-document pairs. We use the principal component analysis (PCA) method [23] to extract valuable information as virtual features from the original data since the PCA is a non-parametric analysis. In other words, there are no parameters to tweak and no coefficients to adjust in the entire process. The major steps of the PCA are described as follows.

The PCA is limited to represent the data as a linear combination of its basis vectors. Let 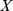 to be the original 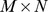 feature matrix obtained from query-document pairs, where 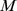 denotes the number of features and 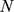 is the number of query-document pairs. 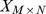 is formed as Equation (1), where each column represents the features of a single query-document pair and each row represents a specific feature of all query-document pairs. Each item 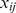 is a feature value of the 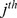 query-document pair for the 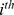 feature. For an example from LETOR 4.0 ^1^, the training data 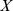 is formed as Equation (2). There are six query-document pairs, and each is represented by a 46-dimension feature vector. From reference [24], we guarantee there exists a linear transformation 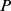 to transform 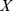 into 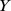 geometrically as Equation (3), where 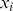 is a column of 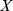 and 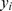 is a column of 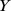. Each row 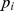 of the matrix 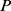 is an eigenvector of 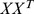 and satisfies the condition as Equation (4). Furthermore we conclude Equation (5), where 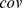 means to compute the covariance of inputted two vectors, and 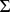 is a diagonal matrix containing the eigenvalues 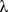 of 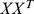. Therefore, we can choose first 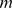 rows (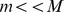) of 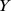 as representative principal components to construct a new matrix 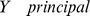 to represent 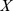, which has been proven to be feasible. Since these 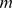 principal components don't have any realistic meanings as initial features, they are called the virtual features of each pair in 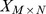. Actually, we intend to choose the optimal parameter 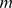, and set 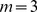 eventually for the following reasons. First, from reference [24], we know that 99% valuable information of initial data is maintained using the first three principal components while 95% using first two principal components. Therefore, preparatory choose of 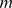 is 3. We also find that the performance can be effectively improved when 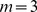; secondly, when we set 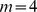 in the experiment, we find that the ranked list of documents and the performance is the same as 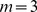. It means that the fourth component contributes a little, and this further validates the conclusion mentioned above in reference [24]; third, in order to show the relationship between virtual features and relevance scores intuitively, we want to plot it and validate the correctness of our assumption, so 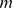 is chosen to be 3.
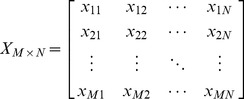
(1)

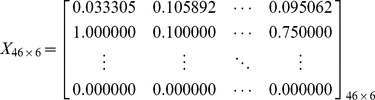
(2)

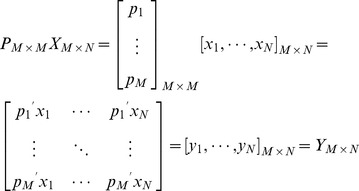
(3)

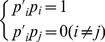
(4)

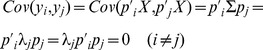
(5)After extracting virtual features by the PCA, we conduct a multivariable logistic regression with the user's relevance feedback. Based on the aforementioned work, we consider a document with 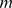 independent variables denoted by the vector 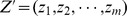, where 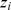 denotes a virtual feature and the conditional probability 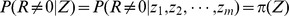, where 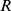 describes the relevance level responded from each user (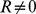 means responded documents are relevant to query). The logit of the multivariable logistic regression model is given by Equation (6), where the logistic regression model is described as Equation (7). Normally, we have 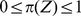.
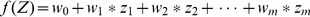
(6)

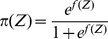
(7)Due to the difficulty in deriving an exact expression of 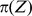, we choose a substitute like Equation(8) to conduct a further regression, where 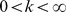, and then derive the Equation(9) as a transformation of Equation (8). Eventually, we perform the linear regression on 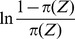 to solve the mentioned problem, and then inversely calculate 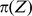. The details of the VFLR algorithm are presented in Algorithm 1. The first 3 lines are used to extract virtual features. From line 4 to l4, it describes the major steps of logistic regression. In line 8 and 10, 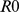 is a temporary vector used to obtain the final relevance indicator vector 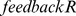, and 0.5 is chosen because that we don't know the exact probability indicating the query is relevant to a document, and also the probability can't be obtained from known training feature matrix, we choose a median of relevant percentage and irrelevant percentage to get relevance indicator, which is an admissive strategy to conduct regression computation.
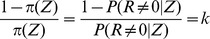
(8)

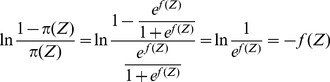
(9)We show an example with an artificial 5 features by 3 query-document pairs to illustrate the entire process. The feature matrix 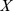 can be formed as (10), and then each row of 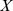 is normalized to a length of 1. Then we obtain 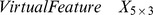 by the PCA, and get vector 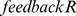 as lines 4 to 14 in Algorithm 1. At last, traditional linear regression (see reference [25]) with inputted parameter 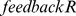 and 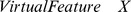 is conducted to output the ranking 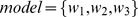.
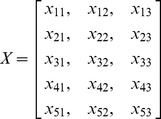
(10)During the test phase, we first have the same manipulation as shown in lines 1 to 3 in the VFLR algorithm, and then use the learned model to compute the ranking score according to Equation(6) by which a ranked list is generated for a query. At last, an evaluation is conducted by comparison with the real relevance from the original dataset. Algorithm 2 shows the details of the testing processes. Line 6 computes the relevance scores of related documents. It means that the relevance score is a linear combination of three virtual features with respective weight. Three public metrics are used to evaluate our proposal from line 10 to 12.

## Experimental Results

### Dataset and Baselines

We use the LETOR 4.0 benchmark datasets to evaluate the VFLR algorithm. LETOR is a package of benchmark datasets for research based on the LEarning TO Rank (LETOR) approach, which not only contains standard features, relevance judgments, data partitioning and several baselines, but also provides evaluation tools and releases the results of all mentioned baselines, such as Ranking SVM [26], RankBoost [27], AdaRank [18], and ListNet [7].

### Algorithm 1 The VFLR algorithm

**Require:** Query-document feature matrix 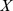, user's relevance feedback vector 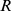 and reduced dimensions 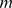 = 3;

**Ensure:** Relevance estimation model 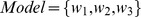.

1. Load feature matrix 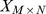 with 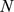 examples and 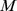 features;

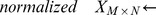
 Normalize Function (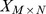); %% Normalize the columns of 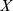 to a length of 1.

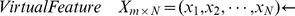
 PCA Function (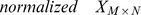, 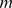);
4. Load feedback vector 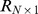;

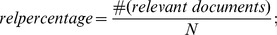
 %% get the percentage of relevant documents and # means to get the total number.
6. **for** each 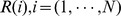 **do**
7. **if**(R(i) =  = 0) **then**

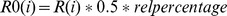
; %% choose a median 0.5 of relevant percentage and irrelevant percentage to get relevance indicator,
9. **else**

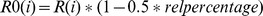
;
11. **end if**

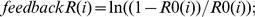
 %% formulate a vector related to the relevance as an input to logistic regression.
13. **end for**

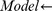
 Regression Function (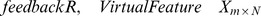);
15. **return** 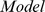

The basic idea of Ranking SVM is to formalize learning to rank as a problem of binary classification on query-document pairs, and then to solve the classification problem using Support Vector Machines. Therefore, constructing the SVM model is equivalent to solving a Quadratic Optimization problem. The major task is to select the best ranking function that minimizes a given loss function with respect to the given instances. In the Ranking SVM algorithm, the linear ranking function is chosen and the parameter c, which allows trading-off margin size against training error, is tuned using the validation set. Like all boosting algorithms, RankBoost operates in rounds, and it assumes access to a separate procedure called the weak learner that, on each round, is called to produce a weak hypothesis. RankBoost chooses a distribution to emphasize different parts of the training data. A high weight assigned to a pair of instances indicates a great importance that the weak learner order that pair correctly. RankBoost trains one weak ranker at each round of iteration, and combines these weak rankers together to obtain the final ranking function. Besides, the document pairs are re-weighted by decreasing the weights of correctly ranked pairs and increasing the weights of incorrectly ranked ones after each round. In the implementation, each weak ranker is defined on the basis of a single feature, and the best weak ranker is selected from the candidates with a proper threshold. ListNet supposes that there is a ranking function assigning ranking scores to all objects, and then makes interchangeable the ranking function and the list of scores. However, there is uncertainty in the prediction of ranking lists. In other words, any ranking list is assumed to be possible, but different ranking lists may have different likelihood values calculated based on the ranking functions. Therefore, it defines the ranking list produced by ranking function a probability distribution, and also defines another distribution based on the ground truth labels. Then it uses cross entropy as difference between the distributions to define the loss function and optimizes it with linear Neural Network as model and Gradient Descent as optimization algorithm based on top k objects probability. Different from existing methods, such as Ranking SVM and RankBoost, which train ranking models by minimizing loss functions loosely related to the performance measures, the AdaRank algorithm, within the framework of

### Algorithm 2 Testing and evaluation

**Require:** Query-document feature matrix 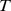, learned 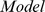 and reduced dimensions 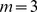; the true relevance vector 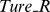;

**Ensure:** Ranking performance measures, e.g. MAP, P@K and NDCG@K.

1. Loading 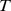;

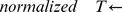
 Normalize Function (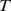); %% Normalize the columns of 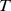 to a length of 1.

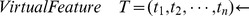
 PCA Function (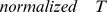, 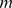); %% 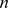 denotes the number of test examples, and 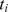 is a column vector representing a document using virtual features.
4. **for** each individual query **do**
5. Compute the relevance score of related document 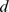 using learned 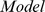;

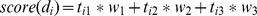
; %% 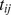 is a element of 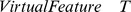 at row 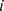 and column 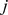.
7. Output a ranked list of documents in descending order by their scores;
8. **end for**
9. %% evaluation
10. Loading 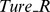;
11. Compute mean average precision (MAP);
12. Compute 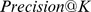 and 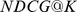
(while 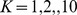);

boosting, minimizes a loss function directly defined on the performance measures. In learning, it repeats the process of re-weighting the training sample, creating a weak ranker, and calculating a weight for the ranker, and finally linearly combines the weak rankers for making ranking predictions. The AdaRank algorithm can iteratively optimize an exponential loss function based on any of IR performance measures. AdaRank-MAP utilizes MAP to measure the goodness of a weak ranker while AdaRank-NDCG directly optimizes NDCG.

From the view of [18], there are three topics related to document retrieval. They are ‘learning to rank’, boosting, and direct optimization of performance measures. Our work in this paper can be viewed as a ‘learning to rank’ method particular for ranking in IR. Different from Ranking SVM, which refers to numerous ranking functions of document features, the VFLR approach utilizes crucial virtual features of documents. Compared to boosting, which predetermines the number of iteration and needs repeatedly re-weighting training data, our VFLR is a non-parametric approach. Besides, it can simultaneously maximize typical IR metrics, such as MAP, Precision and NDCG, other than AdaRank-MAP and AdaRank-NDCG, which only optimize a unique measure and may be propitious to a specific application.

LETOR4.0 is first released in July 2009. It uses the Gov2 web page collection and two query sets from Million Query tracks of TREC 2007 and TREC 2008. We call the two query sets MQ2007 and MQ2008 for short. There are about 1700 queries in MQ2007 with labeled documents and about 800 queries in MQ2008. The relevance judgments from users are given in three levels (highly relevant, relevant, and irrelevant, i.e., 2, 1, 0). Table 1 shows an example from MQ2007. It means that for a query with an id 15 and a document with an id GX009-26-3264567, the label is 2(highly relevant). The 46 features extracted for the query-document pair are {0.997948, 0.000000, 0.250000,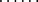,0.000000}.

**Table 1:** An example line in the MQ2007 dataset.

2	qid:15	1:0.997948	2:0.000000	3:0.250000	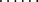	46:0.000000	# docid = GX009-26-3264567

We follow the partitions as LETOR which divides each dataset into five parts, denoted as S1, S2, S3, S4, and S5. In each fold of LETOR, three parts are used for training while one part for validation and the remaining part for test (see Table 2). The training parts are used to learn the ranking model. The test set is used to evaluate the ranking performance of the learned ranking model. The validation set is not used because in the VFLR algorithm, there's no parameter to tune when constructing the ranking model, which is different from other ranking algorithms.

**Table 2:** Data partitions in LETOR.

Folds	Training set	Validation set	Test set
Fold1	(S1,S2,S3)	S4	S5
Fold2	(S2,S3,S4)	S5	S1
Fold3	(S3,S4,S5)	S1	S2
Fold4	(S4,S5,S1)	S2	S3
Fold5	(S5,S1,S2)	S3	S4

### Evaluation Metrics

We use the following metrics [28], [29]: the Mean Average Precision (MAP), Precision at position k (P@k), and Normalized Discounted Cumulative Gain at position k (NDCG@k). They have all been proved to be discriminative and stable in recent years among the TREC community.

The value of MAP is calculated as Equation (11), where 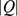 denotes the number of queries and 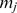 is the number of documents related to query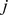, 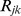 is the set of ranked retrieval results from the top results until one gets the document 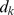,
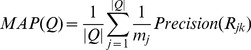
(11)The P@k is thus significant to the commercial search engines. It is calculated as Equation (12), where 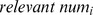 is the number of relevant results among top 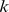 results.
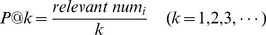
(12)NDCG at the position 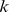 is calculated as Equation (13), where 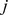 is the position in the document list, 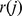 is the relevance score of the 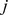
th document in the document list, and 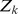 is a normalizing factor. 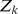 is chosen so that for the perfect list NDCG at each position equals one.
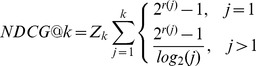
(13)


### Hypotheses Testing

Before evaluating the ranking performance of different algorithms, we first validate the hypotheses over the datasets, i.e., linear relationships exist between the virtual features extracted by the PCA and the relevance of query-document pairs with the consideration that users are always rational to distinguish whether the responded documents are relevant to their queries or not.

**Figure 1:**
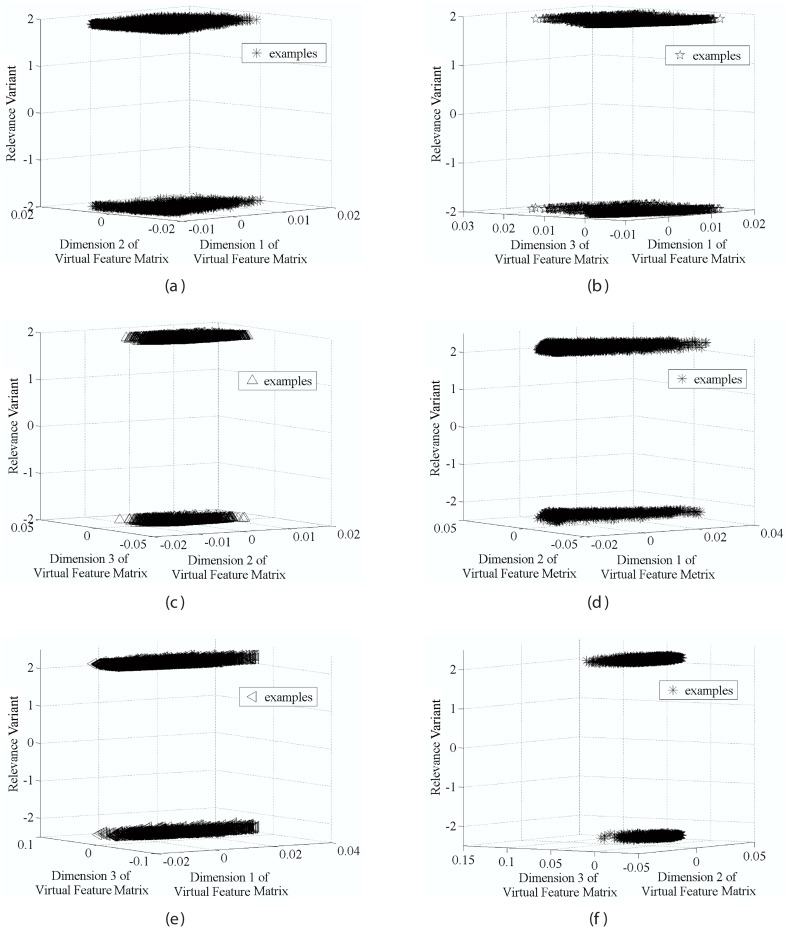
Hypotheses testing on dataset MQ2007 (a)–(c) and MQ2008 (d)–(f).

Firstly, we calculate correlation coefficients as Equation (16) among virtual features, where 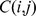 is an element in the covariance matrix 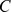 of the virtual feature matrix obtained by the PCA at the position of the 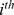 row and 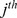 column, calculated as Equation (15), where 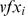 is a column vector of 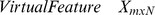 mentioned in Algorithm 1, 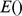 is the mathematical expectation and 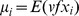. For MQ2007, we obtain 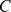 as (14). Therefore each correlation coefficient 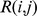, showing the correlation of virtual feature vector 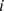 and 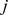, equals or approximates to zero; hence, the virtual features are independent.
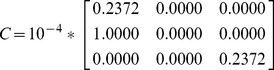
(14)

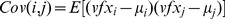
(15)

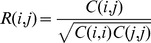
(16)Secondly, we plot the relevance values versus every two virtual features in a 3-D space, respectively. As shown in Fig. 1 (a)–(f), samples represented by virtual features in both datasets are mainly distributed in two opposite surfaces; so we can achieve Equation (17) from Equation (6) and (9), and then get Equation (18) after several ordinary mathematical operations from Equation (17). That means the linear correlation between the virtual features and the relevance values indeed exist in the MQ2007 and MQ2008 datasets. For all the datasets in considered, the statistics support our hypotheses for designing the algorithm.
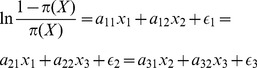
(17)

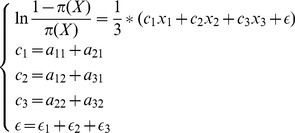
(18)


### Performance evaluation

We start our analysis by evaluating the retrieval quality of the proposed method in this paper, VFLR. We obtain a relevance estimation model from the training dataset. Table 3 and Table 4 show the MAP for the MQ2007 and MQ2008 datasets, respectively. The result of each trial is obtained by averaging partial results obtained from each query. The final average result is obtained by averaging the five trials. The MAP improvements of the VFLR method over the respective baseline, calculated by average MAP, are highlighted in bold.

**Table 3:** MAP for the MQ2007 dataset.

Methods	Trial 1	Trial 2	Trial 3	Trial 4	Trial 5	Avg	Improvement
Ranking SVM	0.4894	0.4573	0.4676	0.4401	0.4680	0.4645	**+9.26%**
RankBoost	0.4891	0.4647	0.4694	0.4384	0.4692	0.4662	**+8.86%**
ListNet	0.4884	0.4565	0.4642	0.4452	0.4716	0.4652	**+9.09%**
AdaRank-MAP	0.4817	0.4510	0.4579	0.4363	0.4618	0.4577	**+10.88%**
AdaRank-NDCG	0.4858	0.4509	0.4579	0.4361	0.4705	0.4602	**+10.28%**
**Proposed VFLR**	**0.5169**	**0.5113**	**0.5082**	**0.4870**	**0.5140**	**0.5075**	**——–**

**Table 4:** MAP for the MQ2008 dataset.

Methods	Trial 1	Trial 2	Trial 3	Trial 4	Trial 5	Avg	Improvement
Ranking SVM	0.4502	0.4213	0.4529	0.5284	0.4950	0.4696	**+16.76%**
RankBoost	0.4666	0.4380	0.4472	0.5342	0.5016	0.4775	**+14.83%**
ListNet	0.4884	0.4565	0.4642	0.4452	0.4716	0.4652	**+17.86%**
AdaRank-MAP	0.4627	0.4232	0.4582	0.5180	0.5198	0.4764	**+15.09%**
AdaRank-NDCG	0.4638	0.4353	0.4560	0.5366	0.5201	0.4824	**+13.66%**
**Proposed VFLR**	**0.5474**	**0.5170**	**0.5334**	**0.5757**	**0.5678**	**0.5483**	**——–**

From the MAP of both datasets, the best individual trial and overall results are always obtained by our VFLR method. As we can see from Table 3, all baseline methods achieve approximative results in the MQ2007 dataset. The worst overall result is obtained by the AdaRank-MAP method (0.4577) and the best among all baselines is achieved by the RankBoost method (0.4662). Our VFLR is the best one (0.5075) compared to existing baseline methods, i.e., the VFLR method improves the MAP by 8.86% at least (relative to the RankBoost method) and 10.88% at most (relative to the AdaRank-MAP method). For the MQ2008 dataset, the AdaRank-NDCG method is the most effective one. As shown in Table 4, the VFLR method achieves prominent improvements (compared to the best baseline AdaRank-NDCG method) in all 5 trials, especially in the first trial. The overall improvement of the average MAP for MQ2008 dataset ranges from 13.66% (relative to the AdaRank-NDCG method) to 17.86% (relative to the ListNet method). Besides, compared to the evaluation on the MQ2007 dataset, the MAP on the MQ2008 dataset entirely exceeds 10% and partially approximates to 18%. These significant improvements on MQ2008 dataset come from the case that the number of relevant documents approximates to that of irrelevant documents in training samples. This case contributes a lot to logistic regression.

**Figure 2:**
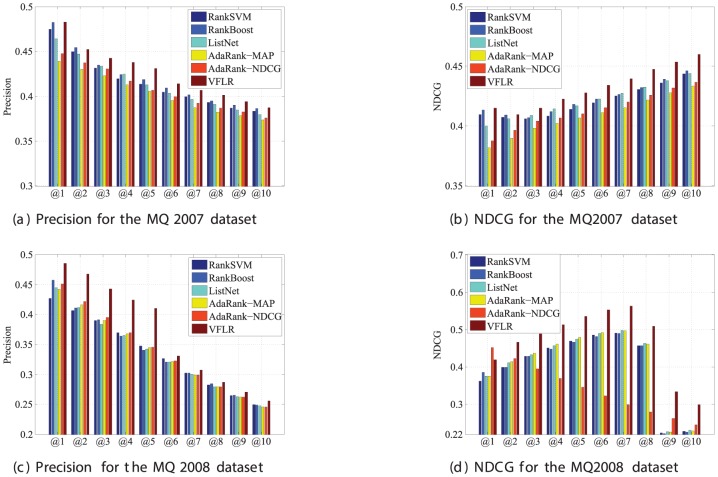
Precision and NDCG for the MQ2007 and MQ2008 datasets.

We also evaluated the VFLR method in terms of the Precision@k and the NDCG@k. Fig. 2 shows the comparison of the NDCG and Precision metrics under existing evaluation methods. As expected, the results demonstrate that the VFLR is the best one. We will use the VFLR to make a comparison with the baselines. Note that the values of NDCG and Precision are the average values of five trials.

For the MQ2007 dataset, the experimental results of baselines are very approximate, especially in terms of Precision. Referring to the NDCG, the VFLR method is able to always provide a slight improvement over the baselines while brings a little bit decrease of the Precision compared to the RankBoost method at the position 2. In any case, impressive improvements are obtained using the MQ2007 dataset. From Fig. 2 (a) and (b), we can see that the VFLR algorithm improves the Precision by 0.06% to 9.88% compared to the RankBoost method at position 1 and the AdaRank-MAP method at position 1, while improves the NDCG by 0.05% to 8.64% compared to the RankBoost method at position 2 and the AdaRank-MAP method at position 1, respectively.

For the MQ2008 dataset, both the Precision and the NDCG, the VFLR algorithm is able to provide a remarkable improvement over the baselines except NDCG@1 (lower than the AdaRank-NDCG method). Similarly, the VFLR algorithm improves the Precision from 1.43% (compared to the RankSVM method at position 6) to 20.54% (compared to the RankBoost method at position 5). Meantime, the VFLR algorithm improves the NDCG by 8.82% at least (relative to the RankBoost method at position 2) and 87.94% (relative to the AdaRank-NDCG method at position 7).

The experimental results indicate that, for both MQ2007 and MQ2008 datasets, the improvements of the VFLR method over all baselines are significant in the MAP while subtle in the Precision and NDCG, especially for MQ2008. We conclude the following observations and main reasons for such an enhancement and improvement. Firstly, Both the MQ2007 and MQ2008 datasets contain a few features, which are extracted basically from textual evidence (such as TF, IDF, and BM25) and additionally from link structures of web pages (such as PageRank, inlink number, outlink number, number of child pages), providing sufficient features to represent query-document pairs. Secondly, the number of examples with disparate relevance in both datasets is close to each other. This phenomenon is propitious to statistical regression.

Furthermore, we plan to investigate some open problems in our future work. (1) Other feedback information from users need to be analyzed and incorporated into the profitable features, e.g., the dwell time of web browsing behaviors [30], [31]. (2) After extracting sufficient valuable features, we can further predict the user's interests and improve the ranking performance. (3) Some statistical approaches may be utilized to perform the sensitivity analysis so as to select the most significant features.

## Supporting Information

The VFLR algorithm is written using Matlab and is available upon request from the author.
